# Effectiveness and safety of acupuncture for treating trigeminal neuralgia associated anxiety and depression: A protocol for systematic review and meta-analysis

**DOI:** 10.1097/MD.0000000000028631

**Published:** 2022-01-21

**Authors:** Ning Luo, Rongrong Li, Yiyi Wang, Yunfan Xia, Jing Sun, Linfang Zhao, Chao Sun, Jiemin Sun, Jianqiao Fang

**Affiliations:** a The Third Clinical Medical College of Zhejiang Chinese Medical University, Hangzhou City, Zhejiang Province, China; b The Third Affiliated Hospital of Zhejiang Chinese Medical University, Hangzhou City, Zhejiang Province, China; c Community Health Service Center of Puyan Street, Binjiang District, Hangzhou City, Zhejiang Province, China.

**Keywords:** acupuncture, anxiety, depression, meta-analysis, protocol, trigeminal neuralgia

## Abstract

**Background:**

Patients with trigeminal neuralgia (TN) often develop a terrible fear of triggering pain, which may lead to anxiety and depression, exerting a negative effect on their quality of life. This protocol is carried out to comprehensively explore the effectiveness and safety of acupuncture for treating anxiety and depression induced by TN.

**Methods:**

Randomized control trials involving acupuncture for treating patients with anxiety and depression caused by TN will be searched in eight electric databases, including PubMed, Web of Science, EMBASE, Cochrane Central Register of Controlled Trials, Chinese National Knowledge Infrastructure, Chinese Biomedical Literature Database, Wanfang Database and Technology Periodical Database (VIP). In addition, studies that were reported in Chinese or English will be considered. Studies selection, data extraction and risk of bias assessment of the included studies will be conducted independently by two reviewers. Quality of the included studies will be performed according to the Cochrane Risk of Bias tool. Meanwhile, the level of evidence for results will be assessed by using the Grading of Recommendations Assessment, Development, and Evaluation method. The primary outcomes will be the Hamilton Anxiety/Depression Scale or Zung Self-Rating Anxiety/Depression Scale, secondary outcomes will be the visual analog score, numerical rating score, SF-36, and adverse events. All analyses will be conducted by using the RevMan software V5.3.

**Results:**

A high-quality synthesis of current evidence of acupuncture for TN patients associated with anxiety and depression will be provided in this study.

**Conclusion:**

This systematic review will offer comprehensive evidence of acupuncture on specific outcomes induced by TN and TN-related anxiety and depression.

**Trial registration:**

PROSPERO registration number: CRD42020219775.

## 1 Introduction

Trigeminal neuralgia (TN) is a very painful neurological condition with severe, stimulus-evoked, short-lasting stabbing pain attacks in the face which could be triggered by talking, washing, brushing or touching.^[1,2]^ According to etiology, there are 3 categories: idiopathic trigeminal neuralgia, classic trigeminal neuralgia, and secondary trigeminal neuralgia. Given that the classification method has been endorsed by both the International Headache Society and the International Association for the Study of Pain.^[3–5]^ Population-based European studies have found that the incidence of TN ranges from 12.6 to 27.0 per 100000 person-years.^[6,7]^ An epidemiological study demonstrated that with the increase of anxiety, depression, and poor sleep in patients with TN, highlighting the impact of disease on mental health.^[8]^ High pain intensity and ineffective medicine treatment were significant risk factors for anxiety and depression in patients with TN.^[9]^ Therefore, it is even known historically as the ‘suicide disease’.^[10]^ Significantly, it is very important to find more effective treatments to alleviate the anxiety and depression symptoms of patients with TN.

Currently, TN, anxiety and depression are often treated independently. The most frequently prescribed drug for TN remains to be carbamazepine,^[1,11,12]^ which also caused significant intolerable side effects, mainly affecting the central nervous system, such as dizziness, poor concentration, and ataxia.^[12–15]^ Given insufficient efficacy, poor tolerability, and adverse reactions, surgery was the first choice for referral, such as microvascular decompression, ablative treatments, gamma knife surgery. However, complications of surgical treatments are inevitable, such as facial hypoesthesia, masticatory atonia, auditory perceptual disorders, etc.^[16–19]^ Pharmacotherapy and psychotherapy, the main treatments for anxiety and depression, play an important role in improving these distressing emotions.^[20]^ Nevertheless, anxiolytics and antidepressants may bring a variety of adverse impacts such as headaches, addiction and suicide, etc.^[21]^ Besides, the psychological intervention available to patients is also limited due to the lack of providers and financial resources.^[22]^ In summary, searching for an alternative treatment that is effective, cheaper, and safer is warranted, purpose to relieve anxiety and depression while alleviating the pain intensity of TN.

As one of the important methods in traditional Chinese medicine, acupuncture has a long history of treating TN, studies also demonstrated that the effectiveness of acupuncture in relieving the pain intensity of TN.^[23–25]^ Additionally, robust evidence identified the effectiveness of acupuncture in treating anxiety and depression,^[26,27]^ but the effectiveness of acupuncture for anxiety and depression caused by TN is still remains largely uncertain and the relevant evidence is limited.

To our knowledge, there are no Systematic Reviews and Meta-analysis to evaluate whether acupuncture therapy is safe and effective in treating anxiety and depression caused by TN. Therefore, the first produce a credible protocol will be carried out to confirm it. Furthermore, we expect that the results will provide a robust evidence to remedy the inadequate evidence in this field.

## 2 Methods

### 2.1 Study registration

This systematic review protocol has been registered on PROSPERO (https://www.crd.york.ac.uk/prospero/#myprospero) and the egistration number is CRD42020219775. Protocol for this review will be strictly carried out and reported in accordance with the Preferred Reporting Items for Systematic Reviews and Meta-Analysis Protocols statement guidelines.^[28]^

### 2.2 Ethics and dissemination

Because of the publications included in this study do not involved patients’ privacy, and the main data in this study will be extracted from those published studies. Given that ethics approval will not be required. In addition, the results of this Meta-analysis will be published in a peer-reviewed journal and presented at an international academic conference for dissemination.

### 2.3 Criteria for considering studies

#### 2.3.1 Type of studies

We will evaluate the studies according to the criteria of participants, interventions, comparisons, outcomes. Only randomized controlled trials (RCTs) comparing acupuncture to either placebo or sham, no treatment, conventional therapies or Chinese herbal medicine for anxiety and depression in patients with TN will be included. Because the focus of this study is to assess the sentiments changes of anxiety or depression after acupuncture, the outcomes related to evaluating anxiety or depression are required in the trials. Indeed, only studies reported in English and Chinese will be included. To avoid the risk of bias, using incorrect randomization methods will be excluded, such as quasi-RCTs, crossover trials, and cluster-RCTs. Additionally, other designs such as case reports, comments, cohort studies, animal experiments, and reviews will also be excluded.

#### 2.3.2 Types of participants

Participants diagnosed with TN will be included, and the diagnostic criteria is based on the ‘European Academy of Neurology Guideline on Trigeminal Neuralgia’.^[13]^ All TN patients feeling anxious and depressed will be included. No limited will be applied in terms of age, gender, nationality and ethnicity. However, those patients with epilepsy, head injury, or other related neurological diseases will be excluded.

#### 2.3.3 Types of interventions

The interventions under consideration must involve needle insertion at acupuncture points, pain points or trigger points, and had to be described as acupuncture. Studies evaluating the following treatments, including body acupuncture (manual acupuncture or electroacupuncture), auricular acupuncture, scalp acupuncture, warm needle acupuncture, plum blossom needling and fire needling, will be considered. In addition, studies involving acupuncture as the monotherapy or combined with other treatments identical to the control group will also be included in this review. However, other methods of stimulating acupuncture points without needle insertion, such as moxibustion, laser stimulation, massage or transcutaneous electrical nerve stimulation, will be excluded.

#### 2.3.4 Types of comparator(s)/control

The inclusion of the comparator mainly included sham or placebo acupuncture intervention such as non-penetrating, sham needle or superficial needling at non-acupuncture points will be considered. Besides, waiting list control, western medicine, moxibustion, massage and psychological intervention will also be taken into account. However, studies comparing between different types of acupuncture therapies, such as including only compared different manipulation forms or acupoint-selection methods of acupuncture, will be excluded.

All inclusion and exclusion criteria for considering studies for this review were summarized in Table 1.

**Table 1:** Inclusion and exclusion criterion for considering studies for this review.

Study selection	Inclusion	Exclusion
Studies	1. RCTs involving acupuncture against another treatment or placebo/sham in patients with TN.2. Studies that the term of “randomization”was mentioned.3. Outcomes related to evaluate anxiety or depression is required.4. Studies that were reported in Chinese or English.	1. Incorrect randomization methods.2. Other designs (such as in vivo, in vitro, case report and non-RCTs).3. trials without control group.
Participants	1. All TN patients with anxiety and depression who received acupuncture therapy.2. There are no restrictions on age, gender, nationality and ethnicity.	1. Not meet diagnostic criteria for TN.2. Not accompanied by anxiety or depression.
Interventions	1. Body acupuncture, including MA or EA.2. Auricular acupuncture.3. Scalp acupuncture.4. Warm needle acupuncture.5. Plum blossom needling.6. Fire needling.7. Acupuncture combined with other therapies.	1. Moxibustion.2. Laser stimulation.3. Massage.4. Transcutaneous electrical nerve stimulation.
Comparators or control	1. Sham/placebo acupuncture.2. Waiting list.3. Western medicine.4. Moxibustion.5. Massage.6. Psychological intervention.	1. Comparing different acupoints.2. Different forms of acupuncture.

### 2.4 Outcome measures

#### 2.4.1 Primary outcomes

To be considered for inclusion, trials must have evaluated the following primary efficacy outcome measures from the beginning of acupuncture treatment, including the level of anxiety or depression measured by qualified scales, such as the Hamilton Anxiety/Depression Scale, Zung Self-Rating Anxiety/Depression Scale.

#### 2.4.2 Secondary outcomes

The secondary outcome measures include: (1) The change in pain symptoms of TN from baseline to the endpoint, based on the visual analog score, numerical rating score or any other validated scale for the assessment of overall TN symptoms when available, (2) The quality of life based on measurement with a validated scale, such as the Short Form 36 Health Survey (SF-36) and (3) Adverse events.

### 2.5 Search strategies

Two independent researchers will search the following eight electronic databases from inception to June 2021, which including PubMed, Web of Science, EMBASE, the Cochrane Central Register of Controlled Trials, Chinese National Knowledge Infrastructure, Chinese Biomedical Literature Database, Wanfang Database and Technology Periodical Database. We will use medical subject headings and text words related to ‘trigeminal neuralgia’ and ‘acupuncture’ and ‘randomised controlled trials’ for publications searching. The specific search strategy for PubMed will be taken as an example, which will be shown in Table 2. Similar search strategies will be used in other electric databases. No language restriction will be applied. In addition, the reference lists of previous systematic reviews will be examined to ensure the quantity and accuracy of the included studies.

**Table 2:** Search strategy used in PubMed database.

NO.	Search items
#1	Randomized controlled trial [pt]
#2	Controlled clinical trial [pt]
#3	Randomized controlled [tiab]
#4	Randomized [ti, ab]
#5	Controlled [ti, ab]
#6	Clinical trials [MeSH]
#7	Randomly [ti, ab]
#8	Trial [ti]
#9	#1 OR #2 OR #3 OR #4 OR #5 OR #6 OR #7 OR #8
#10	Humans [MeSH]
#11	#9 AND #10
#12	Trigeminal Neuralgia [MeSH]
#13	Ticdouloureux [MeSH]
#14	Trigeminal Nerve [ti, ab]
#15	Prosopalgia [MeSH]
#16	#12 OR #13 OR #14 OR #15
#17	(Acupuncture therapy) OR (Electroacupuncture therapy) [MeSH]
#18	Acupuncture OR Electroacupuncture OR (Manual acupuncture) OR (Acupuncture and Moxibustion) OR (Auricular acupuncture) OR (warm needling) OR (Acupoint) [ti, ab]
#19	#17 OR #18
#20	Depression [MeSH]
#21	Depressions[ti, ab]
#22	Depressive [ti, ab]
#23	Anxiety [MeSH]
#24	Hypervigilance [ti, ab]
#25	Nervousness [ti, ab]
#26	#20 OR #21 OR #22 OR #23 OR #24 OR #25
#27	#11 AND #16 AND #19 AND #26

Besides, the authors will also search the relevant studies from clinical trial registries, like the WHO International Clinical Trial Registry Platform, Chinese clinical registry and ClinicalTrials.gov, Google scholar and ongoing trials with unpublished data. We will contact the corresponding author of articles if there are any questions.

### 2.6 Study collection

**Figure 1:**
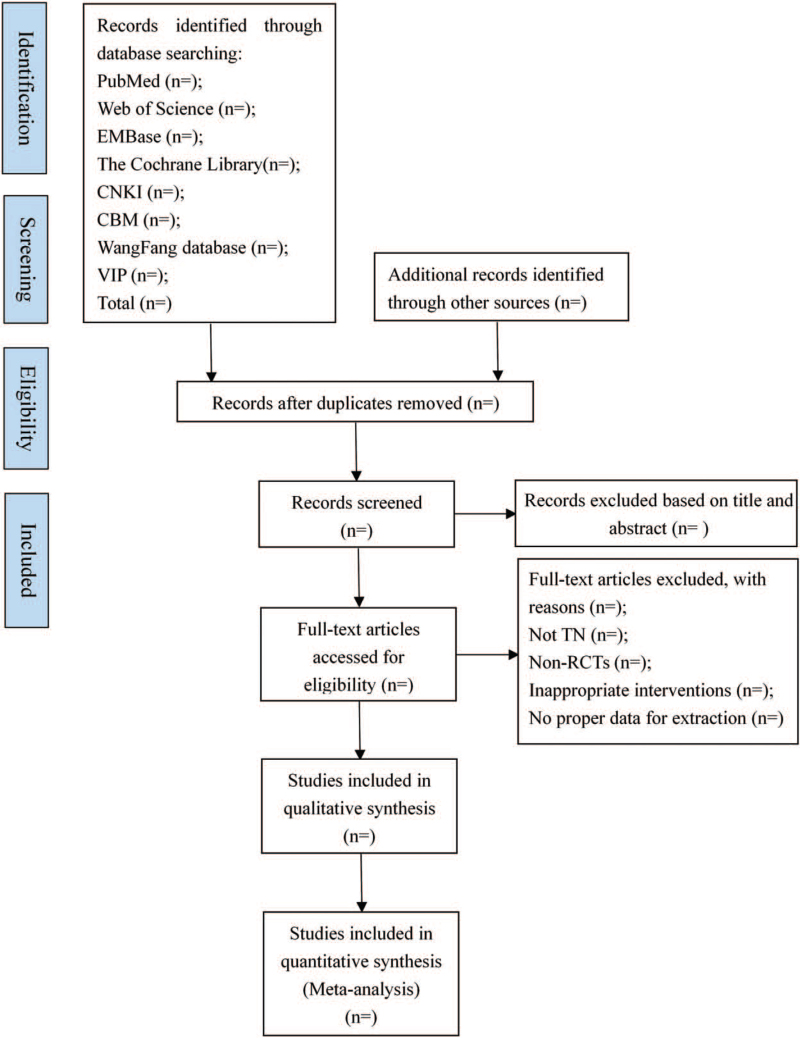
Flow diagram of the study selection process.

Two independent reviewers (NL and RRL) will evaluate the title and abstract of all studies for possible candidates. Any duplicate studies will be removed. After the first selection is made, the full-text copies of all eligible studies will be downloaded for re-evaluation. Once the reviewer is uncertain about the eligibility of any study, its full text will be obtained to re-examine. In addition, possible conflicts will be resolved by discussion, which will also include a third reviewer (YYW or YFX). The specific process of studies screening will be displayed in Preferred Reporting Items for Systematic Reviews and Meta-Analyses. The flowchart illustrating this study is presented in Figure 1.

### 2.7 Data extraction and processing

The data will be extracted by two reviewers independently from the selected reports or studies and fill in the designed data extraction form. We will obtain data for general information (author, date of publication, country and region, etc), participants, diagnostic criteria for TN with anxiety and depression, methods, interventions, outcomes, results, adverse events, conflict of interest, ethical approval and other information. Moreover, we will contact the corresponding authors by email for further information when the reported data is not sufficient. Any disagreement will be resolved by discussion between the two authors and further disagreements will be arbitrated by the third author (JQF).

### 2.8 Risk of bias (quality) assessment

Two authors (NL and RRL) will assess the risk of bias via the Cochrane Collaboration's tool for risk of bias assessment for all included studies,^[29]^ covering randomisation, concealment allocation, blinding of participants and personnel, blinding of outcome assessors, selective outcomes reporting, incomplete outcome assessors and other sources biases. The judgment on these items will be classified into three levels, ‘low risk of bias’ ‘high risk of bias’ or ‘unclear risk of bias’. In addition, we will grade the quality of the evidence based on the Grades Profiler as the Grading of Recommendation, Assessment, Development, and Evaluation system.^[30]^ The assessments will be regarded into four levels, ‘high quality’ ‘moderate quality’ ‘low quality’ and ‘very low quality’.^[31]^ The graphical presentation of assessment of the risk of bias will be generated by RevMan V.5.3. Besides, the conflicts or any discrepancies will be resolved via discussion or judged by another reviewer (JQF) to achieve the consensus.

### 2.9 Measurement of the treatment effects

The Review Manager software 5.3 (V.5.3) will be applied to perform statistical analysis. Methods for measuring treatment efficacy will vary according to the type of data. For those continuous results, the mean difference (MD) or standardized MD will be used for effective evaluation. Meanwhile, the dichotomous outcomes, usually described to the safety or adverse events, will be analyzed by using a risk ratio with 95%CI.

### 2.10 Dealing with missing data

To obtain the missing data, we will attempt to contact the corresponding author firstly by sending an E-mail. However, in case of no reply from the corresponding author or missing data cannot be supplied, we will estimate the missing data as follows. First, if an intention to-treat analysis were performed in the included studies, we will use the intention to-treat data instead of missing data as the first option. Besides, for consecutive missing data, re-calculate MD and SD values will be executed to further analysis.^[32]^ Furthermore, if possible, sensitivity analyses will be performed to assess how sensitive the results are to reasonable changes in the assumptions that are made. The potential impact of missing data on the final outcome of the review will be addressed in the discussion.

### 2.11 Assessment of heterogeneity

Statistical heterogeneity will be assessed with the *I*^2^ statistical test. An *I*^2^ less than 50% indicates a low level of statistical heterogeneity, subsequently, the fixed-effect model will be used for data analysis. However, if *I*^2^ test 50% or more will be considered as substantial heterogeneity, and sensitivity analysis and subgroup analysis will be executed to find the possible reasons from both clinical and methodological perspectives.

### 2.12 Data synthesis

Data synthesis will be carried out by using Review Manager software 5.3 (V.5.3). The level of heterogeneity contributes the selection of model used in the analysis. The fixed-effects model will be used in the little statistical heterogeneity studies, otherwise, the random-effects model will be used (the I^2^ is or more than 50%). nevertheless, meta-analysis will not be performed if there is considerable heterogeneity in the trials.

### 2.13 Subgroup analysis

Subgroup analysis will be executed if data is available. To detect possible heterogeneity of the results, subgroups analysis will be performed about the following four aspects: (1) sex, (2) Chinese studies vs other countries’ studies, (3) acupuncture vs controls or different type of sham acupuncture, (4) length of treatment differences. In addition, subgroup analysis will also be conducted if any significant covariates contribute to the heterogeneity.

### 2.14 Sensitivity analysis

To test the robustness of the review conclusions, a sensitivity analysis will be performed for the primary outcomes. Several decision nodes, such as sample size, methodological weakness, and missing data, will be considered. The results of the sensitivity analysis will be presented in summary tables. The risk of bias in the review process as indicated by the results of the sensitivity analysis will be discussed.

### 2.15 Reporting bias

Reporting bias will be explored by constructing funnel plots if there are at least 10 trials included in Meta-analysis. Otherwise, Egger test will be performed by using STATA V. 15.1 software.

### 2.16 Patient and public involvement

No patient or public will be involved in our study directly. We only use data that existed in published studies.

## 3 Discussion

TN is a rare neuropathic disorder with excruciating facial pain. The unpredictable pain attacks may result in anxiety and depression, and people's quality of life is further seriously affected. Previous studies have ascertained that patients affected by TN are at a higher risk of developing depression and anxiety than those with atypical facial pain.^[33]^ Besides, anxiety and depression can also exacerbate the pain intensity and decrease the therapeutic effect, simultaneously.^[9]^ However, the psychological consequences of TN, especially depression and anxiety, have received rarely attention. Herein, more effective treatment is warranted for the patients with TN associated anxiety and depression in order to improve the efficacy of treatment and patients’ quality of life.

As a major part of traditional Chinese medicine, acupuncture may have the potential to manage the symptoms of TN, anxiety and depression simultaneously. Unfortunately, there are no robust evidence to ascertain the therapeutic effect of it. Hence, we will perform the first comprehensive Systematic Review and Meta-analysis to assess the efficacy and safety of acupuncture in treating TN associated with anxiety and depression. Based on rigorous study design and accurate evaluation of the studies, we expect that the review will provide robust evidence of acupuncture for treating anxiety and depression accelerated by TN.

## Acknowledgments

The authors would like to express their gratitude to all the advisors of this study.

## Author contributions

NL and RRL conceived and designed the idea, NL, RRL, YYW, and YFX registered the protocol review in the PROSPERO database and drafted the manuscript. LFZ, JMS and CS designed the search strategy. And all authors were involved in the interpretation of the study findings. JQF and JS revised the manuscript. All authors have critically reviewed, provided intellectual input to the manuscript, and approved the final version of the manuscript.

**Data curation:** Yunfan Xia, Jiemin Sun.

**Formal analysis:** Ning Luo.

**Funding acquisition:** Jianqiao Fang.

**Methodology:** Yunfan Xia, Jing Sun, Jiemin Sun, Jianqiao Fang.

**Resources:** Linfang Zhao.

**Software:** Jing Sun, Linfang Zhao, Chao Sun.

**Supervision:** Chao Sun.

**Writing – original draft:** Ning Luo, Rongrong Li, Yiyi Wang.

**Writing – review & editing:** Ning Luo, Rongrong Li, Yiyi Wang, Jianqiao Fang.
